# SIM-PCSR: Key-Layer Complementary Enhancement for UAV RGB-IR Small-Object Detection

**DOI:** 10.3390/s26123806

**Published:** 2026-06-15

**Authors:** Jun He, Yunpu Yang, Jun Li

**Affiliations:** Electronic Information Engineering Program, College of Physics and Electronic Engineering, Sichuan Normal University, Chengdu 610066, China; 2023070612@stu.sicnu.edu.cn (J.H.); 2024070851@stu.sicnu.edu.cn (Y.Y.)

**Keywords:** UAV object detection, RGB-IR fusion, small-object detection, multimodal object detection, cross-modal complementarity, YOLOv8

## Abstract

Unmanned aerial vehicle (UAV) red–green–blue–infrared (RGB-IR) object detection is important for traffic monitoring, security surveillance, and urban management, but remains challenging because aerial targets are often small, densely distributed, and affected by complex backgrounds. In addition, RGB and infrared (IR) modalities contribute unequally under different imaging conditions, making simple feature concatenation or indiscriminate middle-layer fusion insufficient for stable cross-modal utilization. To address this problem, this paper proposes Selective Interaction Mechanism and Prefiltering Complementary Spatial Refinement (SIM-PCSR), a key-layer complementary enhancement method for UAV RGB-IR small-object detection. The proposed method decomposes cross-modal modeling into two stages. SIMAdapter first performs selective interaction on the small-object-sensitive P3 layer before fusion, suppressing redundant responses and enhancing potentially complementary modal evidence. PCSR then refines the fused representation through prefiltering, modal selection, and local window residual refinement, injecting reliable complementary information into the key-layer fused feature in a controlled manner. Experiments on the DroneVehicle dataset show that SIM-PCSR achieves 85.323 mean average precision (mAP)_50_ and 63.572 mAP_50:95_, improving the Fixed Middle Fusion baseline by 0.523 and 0.751 percentage points, respectively. These gains correspond to relative improvements of 0.62% and 1.20% over the baseline. Module ablation, position ablation, repeated-seed evaluation, category-wise analysis, scale-wise analysis, and qualitative visualization jointly demonstrate that explicit selection and organization of cross-modal information can improve UAV RGB-IR small-object detection under modality imbalance and background interference.

## 1. Introduction

Object detection is a fundamental task in computer vision and has been widely applied in autonomous driving, robotic perception, medical assistance, and industrial inspection [1]. With the advantages of flexible deployment, low operating cost, and wide-area observation, unmanned aerial vehicles (UAVs) have become increasingly important in fire inspection, traffic monitoring, urban management, and security surveillance. These applications have also made UAV object detection an important research topic in visual perception [2,3]. However, compared with conventional ground-view scenarios, UAV imagery is usually characterized by small object sizes, sparse object distributions, complex backgrounds, and large scale variations [4,5]. Under challenging conditions such as cloudy weather, fog, and nighttime illumination, the extent to which objects can be discriminated in visible-light images may further degrade, making reliable object detection more difficult.

Infrared (IR) images describe objects through thermal radiation responses. Compared with visible RGB images, which provide rich texture and color details, IR images are visually less detailed but often more robust under weak illumination, nighttime, and complex lighting conditions [6,7]. Since RGB and IR modalities are naturally complementary in terms of information type and operating condition, RGB-IR multimodal detection has been widely regarded as an effective way to improve robust UAV perception [8,9]. Nevertheless, modal complementarity does not imply that simple fusion is always effective. In overhead small-object scenarios, the objects themselves contain limited discriminative information, while background regions occupy most image pixels. Direct concatenation or indiscriminate fusion of dual-modal features may introduce a large amount of detection-irrelevant redundancy from the weaker modality, thereby diluting truly useful cross-modal complementary cues [10,11].

Therefore, the key issue in UAV RGB-IR small-object detection is not only whether multimodal information is complementary, but also how cross-modal information can be selectively utilized under modality imbalance, sparse object information, and strong background interference. Existing methods often emphasize stronger global fusion capacity or more complex multi-scale interaction structures, but they provide limited explicit constraints on which cross-modal responses should be preserved and which should be suppressed [12,13,14]. Meanwhile, due to the aerial viewpoint and long imaging distance, objects in UAV scenes usually occupy only a few pixels, which weakens the effectiveness of fine-grained texture and color cues in RGB images [15]. In contrast, the IR modality can capture thermal responses and tends to form more stable object responses in such scenarios. Without an effective feature selection mechanism, simple feature fusion can be dominated by the stronger modality while also importing noise from the weaker modality, making sustained performance improvement difficult. This problem becomes more pronounced for small objects, where useful information is inherently sparse.

Motivated by this observation, this paper does not aim to stack a more complicated multimodal detection framework. Instead, we focus on building a selective cross-modal utilization mechanism for complex interference conditions. To this end, we propose SIM-PCSR, which decouples cross-modal modeling from the perspective of information selection and organization. Specifically, SIMAdapter first performs constrained selective interaction before feature fusion, suppressing irrelevant background responses and highlighting modal information with higher complementary value. PCSR is then introduced after fusion to reorganize and refine complementary information, preserving effective responses while further reducing the bias caused by modality imbalance. In this way, the cross-modal utilization process is changed from indiscriminate fusion to a two-stage process of selection followed by organization, which better matches the coexistence of sparse object information and background noise in small-object UAV scenes.

Experiments on the DroneVehicle dataset [8] show that the proposed method consistently improves detection performance under different settings. In a single-run comparison, SIM-PCSR improves mAP_50:95_ from 62.821 to 63.572. In five repeated runs with different random seeds, the average mAP_50:95_ increases from 62.738 ± 0.131 to 63.071 ± 0.262. Compared with representative single-modal and multimodal methods, SIM-PCSR achieves a better balance between detection accuracy and model complexity. Further module ablation, position ablation, scale-wise analysis, and category-wise analysis jointly support the central claim of this work: under modality imbalance and background interference, explicit selection and organization of cross-modal information can more effectively improve detection quality.

The main contributions of this paper are summarized as follows:

1. We analyze the modality imbalance and background interference problems commonly observed in UAV RGB-IR detection, and argue that the key challenge is not to keep stacking more complex fusion structures, but to selectively organize cross-modal features under sparse object information and noisy backgrounds.
2. We propose SIM-PCSR, a lightweight and pluggable selective cross-modal utilization mechanism that explicitly divides cross-modal modeling into two stages: selection and refinement. SIMAdapter performs constrained selective interaction before fusion to suppress redundant responses, while PCSR reorganizes and refines complementary information after fusion to enhance discriminative features under modality imbalance.
3. Extensive experiments on the DroneVehicle dataset demonstrate that the proposed method achieves stable performance gains under multiple settings while maintaining a favorable accuracy–complexity trade-off. Module ablation, position ablation, repeated-seed evaluation, and scale- and category-level analyses further verify the effectiveness of explicit information selection and organization in modality-imbalanced and background-cluttered scenes.

The remainder of this paper is organized as follows. Section 2 reviews related work. Section 3 presents the proposed method. Section 4 reports the experimental settings and results. Section 5 discusses the findings and limitations. Section 6 concludes the paper and outlines future work.

## 2. Related Work

### 2.1. Single-Modal UAV Small-Object Detection

Object detection in UAV scenarios is affected by small object scales, large viewpoint variations, complex backgrounds, and real-time deployment constraints [2,3]. Single-modal UAV detection research therefore often improves multi-scale representation, lightweight backbone design, and detection heads. Recent work on UAV small-object detection further emphasizes the preservation of high-resolution details under limited computational budgets [15]. These studies show the importance of shallow features for recognizing small targets in aerial imagery.

However, the performance ceiling of single-modal methods is still constrained by the imaging mechanism itself. RGB images rely on texture, color, and contour information, which can degrade under nighttime conditions, weak illumination, haze, or shadow occlusion. IR images are more robust to illumination changes, but they often lack texture details and precise boundary information. Even with stronger feature pyramids or attention mechanisms, a single modality cannot fully avoid perception blind spots caused by its own imaging characteristics.

### 2.2. UAV RGB-IR Benchmarks and Multimodal Fusion

Research on UAV visible–infrared collaborative detection has accelerated with the development of public datasets and baseline models. UA-CMDet introduced the DroneVehicle dataset and proposed an uncertainty-aware cross-modal detection framework, supporting UAV RGB-IR detection from task definition and data benchmarking to method evaluation [8]. Based on this foundation, subsequent studies have shifted from verifying the usefulness of dual modalities to designing more effective cross-modal fusion strategies [9,16].

One representative line of work focuses on stronger global modeling ability. Transformer-based and multi-scale fusion methods introduce long-range interaction, prompt tuning, or adaptive pyramid modeling into visible-thermal detection [9,17,18]. This line of research moves beyond simple concatenation or coarse middle-level fusion, but it also increases the need to balance feature hierarchy, interaction strategy, and fusion efficiency.

Beyond UAV-specific methods, general RGB-IR or multispectral object detection has produced several representative fusion frameworks that are commonly used as cross-modal detection baselines. CFT introduces Transformer-based cross-modal fusion and captures long-range dependencies between visible and thermal features through intra-modal and inter-modal attention [19]. ICAFusion adopts iterative cross-attention to mine global complementary information, while reducing fusion complexity through parameter sharing [20]. DEYOLO starts from the YOLO detection framework and introduces dual feature enhancement to improve detection-oriented complementarity between RGB and infrared features [21]. These methods reflect the shift from simple feature concatenation to attention-driven and detection-oriented fusion. However, they mainly emphasize overall fusion capacity or global cross-modal modeling. The role of constrained complementary enhancement at small-object-sensitive layers in UAV scenes remains less explored.

### 2.3. Cross-Modal Complementary Modeling and Dynamic Fusion

In broader RGB-T and RGB-IR detection research, many methods use explicit interaction modules to improve cross-modal feature use. Cross-modality interactive attention, cyclic fuse-and-refine blocks, guided attentive feature fusion, and locality-guided cross-modal aggregation are representative examples [12,13,14,22]. Their common goal is to reduce modality mismatch, suppress redundant responses, and deliver complementary information to useful feature locations.

Modality imbalance and dynamic modality weighting have also received increasing attention. Illumination-aware fusion, probabilistic ensembling, and modality-imbalance modeling explicitly consider that the reliability of different modalities varies across scenes [10,11,23]. These methods treat multimodal detection as a controlled interaction problem rather than a simple accumulation of information from two sensors.

However, most dynamic fusion studies focus on global weight adjustment, region-level alignment, or multi-scale feature enhancement. Their main objective is to improve overall cross-modal fusion capacity. For UAV small-object detection, local details and fine-grained complementary cues in shallow high-resolution features are often more critical. If cross-modal modeling is not constrained according to the feature distribution of small-object-sensitive layers, redundant responses and dominant-modality effects may weaken the fusion gain.

### 2.4. Visible–Thermal Tiny- and Small-Object Detection

Recent benchmark studies further demonstrate that small objects are not a marginal issue in visible–thermal detection, but a central bottleneck. In aerial and remote-sensing detection, datasets and benchmarks such as DOTA and DIOR show large scale variation and complex backgrounds [4,5]. High-resolution small-object detectors further emphasize the difficulty of preserving local details when target pixels are sparse [15]. These settings share several difficulties, including limited target pixels, skewed scale distributions, and insufficient adaptation of existing algorithms to dense small objects.

Existing studies have advanced RGB-IR detection from multiple perspectives, including dataset construction, global fusion design, modality calibration, dynamic weight allocation, and tiny-object benchmarking. However, many methods still emphasize global fusion, broad multi-scale interaction, or dynamic weighting across wide feature ranges. For UAV RGB-IR small-object detection, the shallow high-resolution layer is important because it preserves local cues for small and dense targets. This leaves room for a more constrained form of complementary enhancement at the small-object-sensitive key layer.

## 3. Materials and Methods

This section describes the proposed SIM-PCSR method. The detector follows a standard backbone–neck–head pipeline. The backbone extracts multi-scale RGB and IR features, the neck fuses and propagates P3/P4/P5 features, and the detection head predicts object categories and bounding boxes. SIM-PCSR keeps this pipeline unchanged and adds two operations only around the P3 fusion path. Before fusion, SIMAdapter selects key P3 tokens, performs bidirectional cross-modal attention, and writes the interacted information back to RGB-P3 and IR-P3 features. After fusion, PCSR uses redundancy prediction, soft modal selection, local window attention, and gated residual injection to refine the fused P3 feature. The P4 and P5 fusion paths, the detection head, and the training objective are kept consistent with the baseline.

### 3.1. Overall Framework

**Figure 1:**
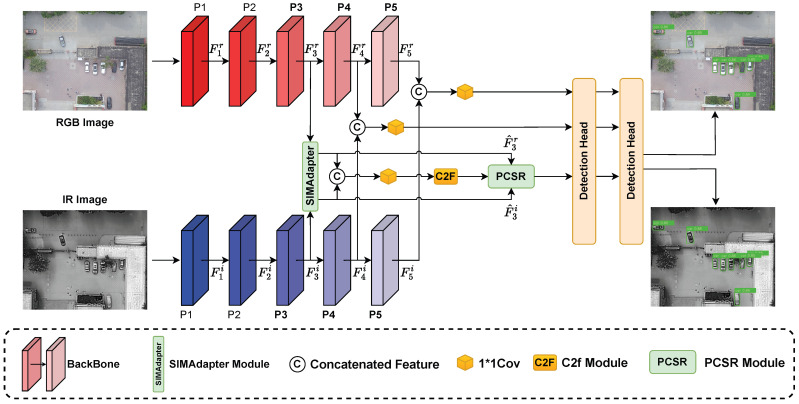
Overall framework of SIM-PCSR. The proposed method introduces SIMAdapter and PCSR only around the P3 key layer of a dual-branch RGB-IR middle-fusion framework to enhance the use of small-object-related cross-modal complementary information.

As shown in Figure 1, the proposed method is built on a dual-branch RGB-IR detection framework and uses YOLOv8s as the base detector [24]. The RGB and IR branches follow the YOLOv8s backbone feature extraction structure, and compatible layers are initialized from YOLOv8s weights to maintain consistent basic detection capability and training settings. Given a paired input, the visible image and infrared image are denoted as $I^{r}$ and $I^{i}$, respectively. The two inputs are fed into their corresponding backbone branches to extract multi-scale features, resulting in $\left\{F_{1}^{r},F_{2}^{r},F_{3}^{r},F_{4}^{r},F_{5}^{r}\right\}$ for the RGB branch and $\left\{F_{1}^{i},F_{2}^{i},F_{3}^{i},F_{4}^{i},F_{5}^{i}\right\}$ for the IR branch. In this paper, $F_{k}$ denotes the feature at the *k*-th scale. Specifically, $F_{3}$ corresponds to the high-resolution P3 layer in the detection pyramid, while $F_{4}$ and $F_{5}$ correspond to P4 and P5, respectively. In the basic framework, middle-level fusion is performed at P3, P4, and P5. The P4 and P5 features are fused using lightweight concatenation followed by a 1×1 convolution, while P3, which is more closely related to local details of small objects, is further equipped with a more refined complementary enhancement path [25].

The proposed modification is not applied to all scales. Instead, it is restricted to the P3 layer. This design is motivated by the fact that P3 preserves higher spatial resolution than the deeper P4 and P5 layers, and therefore more directly carries the edge, contour, and local response information required by small objects in UAV scenes. For this reason, SIMAdapter and PCSR are inserted before and after the basic P3 fusion path, respectively, while the P4 and P5 paths are kept unchanged. This avoids introducing unnecessary complexity across all scales.

From the perspective of information flow, the key-layer processing of SIM-PCSR consists of three steps. First, SIMAdapter acts on RGB-P3 and IR-P3 features and performs selective interaction before fusion, producing the enhanced features ${\hat{F}}_{3}^{r}$ and ${\hat{F}}_{3}^{i}$. Second, the two enhanced features are concatenated and passed through a 1×1 convolution and a C2f (two-convolution cross-stage partial bottleneck) block to generate the basic fused feature $F_{3}^{f}$. Finally, PCSR takes ${\hat{F}}_{3}^{r}$, ${\hat{F}}_{3}^{i}$, and $F_{3}^{f}$ as inputs and performs post-fusion refinement on the key-layer fused representation, producing the final feature ${\tilde{F}}_{3}^{f}$. This refined feature, together with the fused P4 and P5 features, is sent to the subsequent path aggregation network–feature pyramid network (PAN-FPN) and detection head [26].

Therefore, SIM-PCSR is not a new global detector architecture. It is a constrained complementary enhancement path inserted into the most critical shallow scale of a fixed dual-branch middle-fusion framework. This structural arrangement allows the performance gain to be more directly associated with small-object-related representations and also provides a clear basis for the subsequent position and module ablation studies.

### 3.2. SIMAdapter: Selective Cross-Modal Interaction

**Figure 2:**
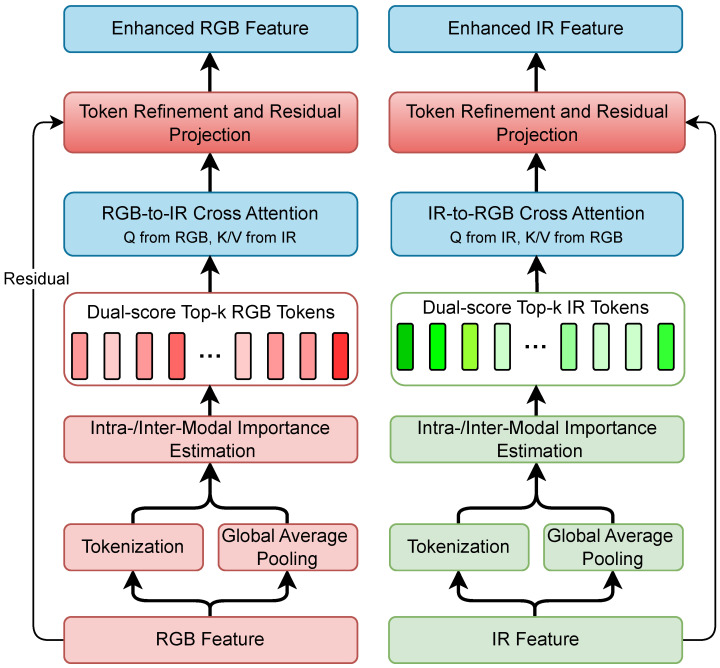
Structure of SIMAdapter. The module first selects key tokens through intra-modal and inter-modal importance estimation, then performs bidirectional cross-modal attention, and finally writes the updated tokens back to RGB-P3 and IR-P3 in a residual manner.

As shown in Figure 2, SIMAdapter is introduced before the basic P3 fusion operation to exchange cross-modal information under a limited computational budget. The name SIMAdapter is derived from Selective Interaction Mechanism Adapter, where SIM denotes the selective interaction mechanism and Adapter indicates that the module is inserted into the key layer of an existing dual-branch detection framework in a lightweight manner. Its design is inspired by the Selective Interaction Module in Signal, which selects important patch tokens from intra-modal and inter-modal perspectives to alleviate background interference in multimodal feature learning. Different from the token selection and global–local alignment framework designed for multimodal re-identification, this work adapts the “select first, interact later” idea to UAV RGB-IR detection. The resulting module acts only on high-resolution P3 features and writes the interacted information back to the dual-modal detection features through residual updates.

In shallow high-resolution features, only a subset of spatial positions contains strong discriminative cues, while most locations correspond to background or low-value responses. If dense interaction is directly performed over the entire spatial feature map, the computational cost increases and the modeling capacity can be distracted by redundant regions. Therefore, SIMAdapter performs cross-modal information exchange only at positions with potentially high responses and high complementary value.

Let $F_{3}^{r},F_{3}^{i}\in R^{C\times H\times W}$ denote the P3 features from the RGB and IR branches. They are first flattened into token sequences, and global semantic descriptors are extracted:

(1) $$T^{r},T^{i}\in R^{N\times C},\;G^{r},G^{i}\in R^{C},\;N=H\times W.$$

To estimate the interaction value of each position, SIMAdapter considers both intra-modal consistency and inter-modal correlation. Specifically, two importance scores are defined by the similarity between normalized tokens and global descriptors:

(2) $${\mathrm{intra}}^{r}\left(j\right)=\frac{\langle {\hat{T}}_{j}^{r},{\hat{G}}^{r}\rangle }{\sqrt{C}},\;{\mathrm{inter}}^{r}\left(j\right)=\frac{\langle {\hat{T}}_{j}^{r},{\hat{G}}^{i}\rangle }{\sqrt{C}}.$$

The IR branch is processed symmetrically. The intra-modal score measures the saliency of a position within its own modality, while the inter-modal score measures its potential association with the global semantics of the other modality.

After obtaining these scores, the module does not rely on a single metric for selection. Instead, it adopts a staged top-*k* strategy to balance intra-modal discriminability and cross-modal complementarity. Candidate positions are first extracted according to intra-modal saliency and inter-modal correlation. The two candidate sets are then merged into a candidate mask, and a joint priority score is constructed:

(3) $$p^{r}\left(j\right)={\mathrm{intra}}^{r}\left(j\right)+{\mathrm{inter}}^{r}\left(j\right)+\lambda M^{r}\left(j\right),$$

where $M^{r}\left(j\right)$ denotes the mask obtained from the union of the two candidate sets, and λ controls the influence of this mask on the final priority. A second top-*k* selection is then applied to the joint score to obtain the final token indices for interaction:

(4) $$T_{\mathrm{sel}}^{r}=\mathrm{Gather}\left(T^{r},I_{\mathrm{final}}^{r}\right),\;T_{\mathrm{sel}}^{i}=\mathrm{Gather}\left(T^{i},I_{\mathrm{final}}^{i}\right).$$

Thus, the retained positions are determined not only by their saliency within the current modality, but also by their potential complementary value to the other modality.

Bidirectional cross-modal attention is then performed only on the selected tokens to exchange complementary information. The interacted tokens are updated by feed-forward mappings and scattered back to their original spatial locations, while unselected positions remain unchanged. Finally, local smoothing and residual injection are used to obtain the enhanced features:

(5) $${\hat{F}}_{3}^{r}=F_{3}^{r}+g_{\mathrm{sim}}^{r}R_{\mathrm{sim}}^{r},\;{\hat{F}}_{3}^{i}=F_{3}^{i}+g_{\mathrm{sim}}^{i}R_{\mathrm{sim}}^{i},$$

where $R_{\mathrm{sim}}^{r}$ and $R_{\mathrm{sim}}^{i}$ denote the residual terms generated by sparse interaction, and $g_{\mathrm{sim}}^{r}$ and $g_{\mathrm{sim}}^{i}$ are learnable scaling factors.

In summary, SIMAdapter follows an importance estimation, key-position selection, sparse interaction, and residual write-back mechanism. It enhances cross-modal complementary responses at the key layer while keeping the overall structure of the original features stable, thereby providing more discriminative inputs for subsequent fusion.

### 3.3. PCSR: Post-Fusion Complementary Refinement

After obtaining the enhanced modal features ${\hat{F}}_{3}^{r}$ and ${\hat{F}}_{3}^{i}$, the model constructs the basic fused feature $F_{3}^{f}$ through channel concatenation, a 1×1 convolution, and a C2f block. Although SIMAdapter improves cross-modal complementarity before fusion, the fused result is still dominated by shared responses, and local complementary information related to small objects may not be fully exploited. To address this issue, we propose PCSR as a post-fusion complementary screening and refinement module. PCSR denotes Prefiltering and Complementary Spatial Refinement. Specifically, the prefiltering stage suppresses redundant dual-modal responses and performs soft modal selection, while complementary spatial refinement injects reliable complementary evidence into the basic fused feature within local windows through a residual path. PCSR does not perform another symmetric global fusion operation. Instead, it introduces a constrained complementary enhancement path on top of the existing fused representation. Reliable modal responses are first selected from the dual-modal features and then injected into the basic fused feature in a residual manner, strengthening key responses while preserving structural stability.

#### 3.3.1. Prefiltering

The features input to the prefiltering stage contain both useful complementary evidence and a large amount of shared background or repetitive responses. If these redundant components are not suppressed, subsequent complementary modeling can be disturbed by invalid information. The prefiltering stage therefore aims to suppress redundant responses and extract reliable complementary evidence. It first decomposes the two modal features into shared and differential representations:

(6) $$F_{\mathrm{sh}}={\hat{F}}_{3}^{r}+{\hat{F}}_{3}^{i},\;F_{\mathrm{df}}=\left|{\hat{F}}_{3}^{r}-{\hat{F}}_{3}^{i}\right|.$$

Based on the shared representation, the differential representation, and the current modal feature, a lightweight mapping consisting of a 1×1 convolution, a 3×3 depthwise convolution, a 1×1 convolution, and a sigmoid activation is used to predict redundancy maps $R^{r}$ and $R^{i}$ for the two modalities. A larger value indicates that the corresponding response is more likely to be redundant and should be suppressed. Cleaner modal features are obtained as follows:

(7) $$F_{\mathrm{clean}}^{r}={\hat{F}}_{3}^{r}⊙\left(1-R^{r}\right),\;F_{\mathrm{clean}}^{i}={\hat{F}}_{3}^{i}⊙\left(1-R^{i}\right).$$

After this suppression, redundant responses are weakened. However, this does not mean that the two modalities should be preserved equally at every location. For subsequent complementary modeling, a more important question is which modal evidence should be retained at each position and how the two modalities should participate in the refinement process. Therefore, shared and differential representations are reconstructed from the cleaned features:

(8) $$F_{\mathrm{sh}}^{\mathrm{cl}}=F_{\mathrm{clean}}^{r}+F_{\mathrm{clean}}^{i},\;F_{\mathrm{df}}^{\mathrm{cl}}=\left|F_{\mathrm{clean}}^{r}-F_{\mathrm{clean}}^{i}\right|.$$

A lightweight mapping composed of a 1×1 convolution, SiLU activation, a 1×1 convolution, and sigmoid activation then predicts modal selection responses $S^{r}$ and $S^{i}$ from the concatenation of $F_{\mathrm{clean}}^{r}$, $F_{\mathrm{clean}}^{i}$, $F_{\mathrm{sh}}^{\mathrm{cl}}$, and $F_{\mathrm{df}}^{\mathrm{cl}}$. These responses are normalized into competitive soft selection weights:

(9) $${\bar{S}}^{r}=\frac{S^{r}}{S^{r}+S^{i}+\varepsilon },\;{\bar{S}}^{i}=\frac{S^{i}}{S^{r}+S^{i}+\varepsilon }.$$

The modal contributions at the same spatial location are therefore constrained to a unified scale, and the selected modal evidence is computed as

(10) $$F_{\mathrm{sel}}^{r}=F_{\mathrm{clean}}^{r}⊙{\bar{S}}^{r},\;F_{\mathrm{sel}}^{i}=F_{\mathrm{clean}}^{i}⊙{\bar{S}}^{i}.$$

To prevent the filtering process from over-perturbing the original features, residual interpolation is used to construct a smooth transition:

(11) $$F_{\mathrm{pref}}^{r}={\hat{F}}_{3}^{r}+g_{\mathrm{clean}}\left(F_{\mathrm{sel}}^{r}-{\hat{F}}_{3}^{r}\right),\;F_{\mathrm{pref}}^{i}={\hat{F}}_{3}^{i}+g_{\mathrm{clean}}\left(F_{\mathrm{sel}}^{i}-{\hat{F}}_{3}^{i}\right).$$

This stage extracts cross-modal complementary evidence through joint redundancy suppression and soft modal selection. The two shared/differential constructions serve different purposes: the representations computed from the original modal features are used for redundancy prediction, while those computed from the cleaned features are used to generate competitive modal selection weights. The corresponding lightweight mappings do not share parameters.

#### 3.3.2. Complementary Window Residual Refinement

After prefiltering, more reliable complementary evidence has been retained in the two modalities, but it has not yet been explicitly injected into the fused representation. Directly performing another global fusion operation would introduce additional computational cost and could weaken the stability of the basic fused feature. Since targets in UAV scenes are highly local, PCSR refines the existing fused feature within local windows. This design reduces computational complexity and better matches the feature distribution of small objects.

**Figure 3:**
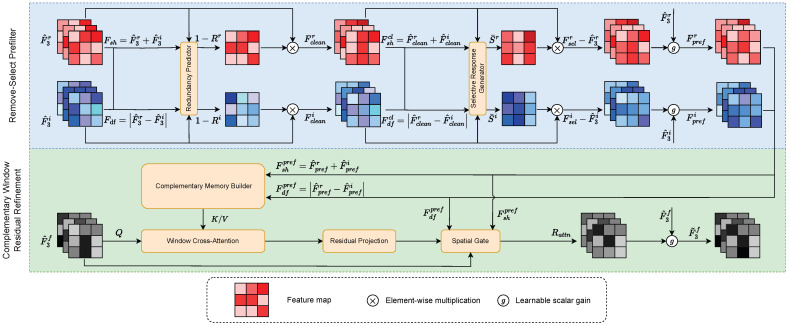
Structure of the PCSR module. PCSR first uses Remove–Select prefiltering to suppress redundant dual-modal responses and generate cleaner complementary evidence, and then injects effective complementary information into the basic fused feature through local window cross-attention and residual refinement. The terms $g_{\mathrm{clean}}$ and $g_{\mathrm{attn}}$ denote learnable gains for the prefiltering and residual refinement stages, respectively.

The structure of the PCSR refinement stage is shown in Figure 3.

To preserve modality-specific information while explicitly encoding consistency and complementarity between the two modalities, a complementary memory representation is first constructed from the prefiltered features:

(12) $$M=\left[F_{\mathrm{pref}}^{r},\;F_{\mathrm{pref}}^{i},\;F_{\mathrm{pref}}^{r}+F_{\mathrm{pref}}^{i},\;\left|F_{\mathrm{pref}}^{r}-F_{\mathrm{pref}}^{i}\right|\right].$$

The basic fused feature $F_{3}^{f}$ is used as the query, and the complementary memory *M* is used as the key and value. Window-based cross-attention is then applied to model complementary context [27]:

(13) $$F_{\mathrm{attn}}=W-\mathrm{CA}\left(F_{3}^{f},M\right).$$

Fixed window partitioning may introduce boundary discontinuities and limit local dependency modeling across neighboring windows. To alleviate this problem, a shifted-window branch is further introduced, and the outputs of the regular and shifted windows are fused to improve the continuity and stability of local modeling.

The attention-enhanced feature is then projected into a candidate residual. Meanwhile, a spatial gate is generated from the basic fused feature, the shared prefiltered representation, and the differential prefiltered representation to constrain where the candidate residual should be injected:

(14) $$G_{s}=\Psi_{g}\left(\left[F_{3}^{f},\;F_{\mathrm{pref}}^{r}+F_{\mathrm{pref}}^{i},\;\left|F_{\mathrm{pref}}^{r}-F_{\mathrm{pref}}^{i}\right|\right]\right).$$

Under the joint modulation of the spatial gate and a global gain, PCSR performs a controlled correction on the basic fused feature. The final injected residual is

(15) $$R_{\mathrm{attn}}=G_{s}⊙\Phi \left(F_{\mathrm{attn}}\right),$$

where Φ(·) denotes a lightweight projection used to generate the candidate residual. The refined feature is then obtained as follows:

(16) $${\tilde{F}}_{3}^{f}=F_{3}^{f}+g_{\mathrm{attn}}R_{\mathrm{attn}},$$

where $g_{\mathrm{attn}}$ is a learnable global gain controlling the residual injection strength. Through this gated residual injection mechanism, reliable complementary information is directed to spatial locations that require local enhancement while the original fused representation remains stable.

In summary, PCSR does not regenerate a new global fusion result. Instead, it injects reliable cross-modal complementary evidence into the existing fused feature in a constrained local manner, thereby enhancing small-object-related responses more effectively.

### 3.4. Detection Head and Optimization Objective

After SIM-PCSR processing, the model obtains the key-layer enhanced fused feature ${\tilde{F}}_{3}^{f}$, which is sent together with the fused features from the other scales to the subsequent PAN-FPN and multi-scale detection head for classification and bounding box regression. The proposed modification focuses on key-layer complementary enhancement and does not redesign the detection head or the training objective. To ensure that the performance gain can be attributed mainly to the module design rather than changes in training strategy, the detection head, loss formulation, and overall training pipeline are kept consistent with the baseline model. We follow the standard YOLOv8 detection objective, where the training loss consists of the classification loss $L_{\mathrm{cls}}$, bounding box regression loss $L_{\mathrm{box}}$, and distribution focal loss $L_{\mathrm{dfl}}$ [24,28]:

(17) $$L=L_{\mathrm{cls}}+L_{\mathrm{box}}+L_{\mathrm{dfl}}.$$

## 4. Results

This section evaluates the proposed SIM-PCSR method on UAV RGB-IR small-object detection. The experiments focus on overall detection performance, the contribution of key components, the stability of the results, and the consistency between the proposed key-layer design and the small-object detection motivation.

### 4.1. Experimental Setup

DroneVehicle is used as the main benchmark dataset [8]. This dataset is selected because it is a publicly available UAV RGB-IR benchmark with paired visible and infrared images, and its aerial viewpoint, dense vehicle distribution, and small target scales are consistent with the problem setting of this study. Since the detector used in this work predicts horizontal bounding boxes (HBBs), while DroneVehicle originally provides oriented bounding box (OBB) annotations, all targets are converted to minimum enclosing horizontal boxes and then transformed into the YOLO detection format. This conversion inevitably introduces additional background regions around tilted vehicles, small objects, and dense targets, making the detection task more challenging. Five categories are retained in all experiments: car, truck, bus, van, and freight car. All main experiments are trained on the official training split and evaluated on the validation split.

For evaluation, mAP_50:95_ is used as the primary metric, and mAP_50_ is also reported. The mAP_50:95_ metric summarizes detection performance over multiple intersection over union (IoU) thresholds and therefore better reflects overall localization quality and detection robustness than mAP_50_ at a single loose threshold. Category-wise and scale-wise analyses are conducted on the same validation set and under the same annotation protocol. The scale groups follow the COCO-style area thresholds: small objects have an area smaller than $32^{2}$, medium objects have an area between $32^{2}$ and $96^{2}$, and large objects have an area no smaller than $96^{2}$.

The implementation is based on the Ultralytics YOLOv8 training framework, with YOLOv8s used as the base detector [24]. Compatible backbone and detection layers are initialized from YOLOv8s weights, while newly introduced multimodal fusion and refinement modules are trained from scratch. Unless otherwise specified, all main methods use an input resolution of 640×640, 150 training epochs, patience of 50, and cosine learning rate scheduling. Multimodal dual-branch experiments use a batch size of 8. Single-modal or lighter comparison methods use the largest stable batch size while keeping the key training settings consistent. All compared methods are evaluated under the same DroneVehicle HBB conversion protocol, official training/validation split, input resolution, and validation metric definitions. All experiments are conducted on a platform equipped with an NVIDIA GeForce RTX 4090 GPU with 24 GB memory. Parameters, giga floating-point operations (GFLOPs), latency, and frames per second (FPS) are reported to compare model complexity and inference efficiency. For SIM-PCSR and its direct baseline, the same data version, image size, augmentation configuration, and validation protocol are used, so that the observed differences can be mainly attributed to the proposed key-layer complementary enhancement strategy.

### 4.2. Main Results

The single-modal results reveal a consistent trend: for Faster R-CNN, RetinaNet, YOLOv5s, and YOLOv8s, the IR input clearly outperforms the corresponding RGB input. In DroneVehicle, RGB images provide richer texture and color information, but the high-altitude overhead viewpoint and long imaging distance make vehicle targets occupy only a small number of pixels, causing fine visual details to degrade. In contrast, the IR modality emphasizes thermal responses and usually provides more stable object representations in small-object scenes. This indicates a clear modality contribution imbalance, suggesting that the core problem is not simply whether multimodal input is useful, but how truly complementary information can be selected and utilized under unequal modal contributions.

The main comparison results are reported in Table 1.

The multimodal comparison further shows that general fusion strategies do not automatically outperform a strong single-modal baseline. Under our unified reproduction setting, DEYOLO, CFT, and ICAFusion obtain mAP_50:95_ values of 60.102, 53.121, and 59.603, respectively, which are comparable to or lower than the strongest single-modal YOLOv8s-IR result. This phenomenon suggests that under modality imbalance and background interference, the benefit of cross-modal fusion depends on effective information selection and organization. Without targeted constraints, additional modal information may not be fully transformed into detection gains.

The proposed SIM-PCSR achieves the best performance among all compared methods, with mAP_50_ and mAP_50:95_ reaching 85.323 and 63.572, respectively. Compared with the strongest external multimodal method, DEYOLO, SIM-PCSR improves mAP_50_ and mAP_50:95_ by 2.367 and 3.470 percentage points. Compared with the strongest single-modal baseline, YOLOv8s-IR, the corresponding improvements are 2.731 and 3.504 percentage points. These gains indicate that the proposed two-stage selection and refinement mechanism can suppress irrelevant responses and strengthen complementary feature representations under modality imbalance.

From the perspective of model complexity, SIM-PCSR maintains a moderate parameter scale of 18.19 M, which is lower than DEYOLO, ICAFusion, and CFT. Therefore, its performance improvement is not mainly caused by a larger model capacity, but by a more effective way of using cross-modal information. Although the computation is higher than that of single-modal baselines, the additional cost remains acceptable for a multimodal detection framework considering the corresponding accuracy gain.

### 4.3. Ablation Study

#### 4.3.1. Module Ablation

To understand the contribution of each component, we progressively introduce different operations of SIM-PCSR on top of the Fixed Middle Fusion baseline. In Table 2, TI denotes top-k selective interaction in SIMAdapter, RT denotes residual transfer to the fused P3 feature, RSP denotes remove–select prefiltering, and WR denotes window-attention refinement. More specifically, RSP includes redundancy prediction and soft modality selection, while WR uses local window attention to inject complementary evidence into the fused representation.

Introducing TI alone does not improve performance. M1 decreases mAP_50:95_ from 62.821 to 62.372, indicating that top-k cross-modal interaction alone is insufficient to form a reliable detection gain. The selected cross-modal responses may contain useful information, but they are not yet stably delivered to the fused P3 representation. After RT is introduced, M2 improves mAP_50:95_ to 62.962 and surpasses the baseline, showing that the interacted information needs an explicit transfer path to affect the final fused feature.

Comparing M3 and M4 further shows that RSP alone does not bring additional gain, while adding WR improves mAP_50:95_ to 63.197. This result suggests that remove–select prefiltering can suppress redundant responses and generate cleaner modal evidence, but it cannot by itself reorganize the retained evidence into the fused representation. In contrast, window-attention refinement directly models local complementary context after fusion and therefore contributes more clearly to detection performance.

The full SIM-PCSR combines RSP and WR and further improves mAP_50:95_ to 63.572, achieving the best result. This indicates that RSP and WR are complementary rather than redundant: RSP provides cleaner and more selectively weighted modal evidence, while WR injects this evidence into the fused feature through local residual refinement.

#### 4.3.2. Position Ablation

The position ablation answers whether the gain of SIM-PCSR comes from generally stacking an additional module or from matching the module with a specific feature layer. Since this work focuses on UAV RGB-IR small-object detection, a complementary enhancement module designed for fine-grained local responses should be more suitable for shallow high-resolution features than for deeper features with compressed spatial details.

As shown in Table 3, moving the same refinement idea from P3 to P5 yields an mAP_50:95_ of 62.748, which is slightly lower than the Fixed Middle Fusion baseline of 62.821. Moving the refinement stage to P4 yields an mAP_50:95_ of 62.825, almost identical to the baseline, while mAP_50_ decreases from 84.800 to 84.264. These results suggest that applying the module at deeper P4 or P5 layers cannot stably release cross-modal complementary information, probably because the lower spatial resolution weakens local details related to small targets. In contrast, placing SIM-PCSR at P3 improves mAP_50_ and mAP_50:95_ to 85.323 and 63.572, respectively.

This result supports the key-layer design of this work. SIM-PCSR is not an indiscriminate enhancement module for arbitrary scales; it is more effective when deployed at P3, where spatial localization information is better preserved and small-object responses are more sensitive.

#### 4.3.3. Complexity Analysis

The complexity analysis evaluates whether the improvement of SIM-PCSR depends on a substantial increase in model size. Compared with the Fixed Middle Fusion baseline, SIM-PCSR increases the parameter count from 17.03M to 18.19M, an increase of approximately 1.16 M. The computation increases from 43.66 GFLOPs to 55.72 GFLOPs. Under this limited complexity increase, mAP_50:95_ improves from 62.821 to 63.572.

The additional parameters mainly come from selective interaction, prefiltering, and local window residual refinement at the P3 key layer, rather than from enlarging the backbone or detection head. The latency increases from 12.54 ms/img to 21.59 ms/img, and FPS decreases from 79.76 to 46.32. This indicates that local window cross-attention and post-fusion residual refinement introduce extra cost, but the model still maintains a practical single-image inference speed. Therefore, SIM-PCSR is better understood as a key-layer complementary enhancement strategy with a limited complexity increase, rather than as a larger detection network.

It should be noted that the latency and FPS values in Table 4 are measured on an NVIDIA GeForce RTX 4090 GPU, rather than on an embedded UAV platform. Therefore, these results provide an initial efficiency reference instead of a complete embedded deployment evaluation. For UAV applications, the current model is more suitable for edge-GPU processing, ground-station analysis, or offline UAV video analysis. Deployment on lightweight airborne devices would require further hardware-specific optimization, such as TensorRT acceleration, pruning, quantization, or more efficient attention designs.

### 4.4. Further Analysis

Beyond the main comparison and ablation studies, we further analyze SIM-PCSR from the perspectives of random-seed stability, category-wise performance, and object scale. These analyses examine whether the improvement is stable across runs, whether it comes from only a few categories, and whether it is consistent with the small-object-oriented motivation.

#### 4.4.1. Multi-Seed Stability

To reduce the influence of training randomness, the Fixed Middle Fusion baseline and SIM-PCSR are repeated five times with different random seeds. As shown in Table 5, we report the mean, standard deviation, and 95% confidence interval for both mAP_50_ and mAP_50:95_. The confidence intervals are estimated using Student’s *t* distribution over five runs. SIM-PCSR improves the average mAP_50:95_ from 62.738 to 63.071, with an average gain of 0.333 percentage points.

The per-seed results in Table 6 further show that SIM-PCSR outperforms the corresponding Fixed Middle Fusion baseline in mAP_50:95_ in all five repeated runs. This reduces the possibility that the improvement under the stricter multi-IoU metric is caused by a single favorable random seed.

Meanwhile, the average mAP_50_ of SIM-PCSR is slightly lower than that of the baseline. This indicates that the benefit of SIM-PCSR is not mainly reflected in coarse detection hits under the loose IoU threshold of 0.50. Instead, the improvement is more evident in mAP_50:95_, which evaluates detection quality across stricter IoU thresholds. This result is consistent with the design of SIM-PCSR, which aims to improve feature discriminability and localization quality through selective prefiltering, local complementary modeling, and controlled residual injection.

#### 4.4.2. Category-Wise AP Analysis

The category-wise analysis investigates whether the overall improvement in SIM-PCSR is dominated by only a few categories. DroneVehicle has an imbalanced category distribution: car instances are much more frequent than other classes, while van, bus, and freight car have fewer samples. If a method only improves the dominant class while degrading rare classes, its overall gain would be less convincing.

Table 7 shows that SIM-PCSR improves mAP_50:95_ on four out of five categories. The largest gains are observed for truck and freight car, with improvements of 1.492 and 1.377 percentage points, respectively. Van also improves by 0.920 percentage points. For the most frequent car class, SIM-PCSR still maintains a slight positive gain, indicating that the method does not sacrifice the dominant category. The bus class shows only a negligible decrease of 0.032 percentage points in mAP_50:95_, which can be regarded as nearly unchanged.

These results indicate that the benefit of SIM-PCSR is not an accidental improvement on a single frequent category. Instead, it shows good adaptability across vehicle categories with different appearances, scales, and sample counts. The gains on truck, van, and freight car further suggest that selective cross-modal complementary modeling helps enhance discriminative representations for more challenging categories.

#### 4.4.3. Scale-Wise Analysis

The scale-wise analysis examines the consistency between the proposed method and the small-object detection motivation. Since SIM-PCSR is designed at the P3 key layer, its direct motivation is to use high-resolution features to select and reorganize cross-modal complementary information related to small objects. If the module did not improve the small-object subset, the key-layer argument would be weakened.

As shown in Table 8, SIM-PCSR obtains positive gains on small, medium, and large objects. For small objects, AP_50_ increases from 26.141 to 27.136, and AP_50:95_ increases from 13.344 to 13.756, corresponding to gains of 0.995 and 0.412 percentage points. The gain under AP_50:95_ is positive but relatively modest. This is mainly because small vehicles in DroneVehicle occupy only a few pixels, and the conversion from oriented bounding boxes to horizontal bounding boxes can introduce extra background regions around tilted vehicles. Under stricter IoU thresholds, even a small localization offset may substantially reduce the IoU of a small box. Dense vehicle layouts, occlusion, and background clutter further increase the difficulty of high-IoU localization for small objects.

Medium and large objects also benefit from SIM-PCSR. In particular, AP_50:95_ for large objects improves by 1.915 percentage points. This larger gain should be interpreted carefully. Large objects provide more pixels and more stable RGB/IR evidence, and their IoU is less sensitive to small absolute localization errors. In addition, SIM-PCSR improves cross-modal complementary representation at the P3 layer, and the cleaner low-level fused representation can propagate through the subsequent feature pyramid to support multi-scale detection. Therefore, SIM-PCSR is a small-object-oriented key-layer enhancement method rather than a scale-exclusive detector. Its benefit is positive on the small-object subset, but improving high-IoU localization for very small and dense targets remains an important direction for future work.

### 4.5. Qualitative Results

**Figure 4:**
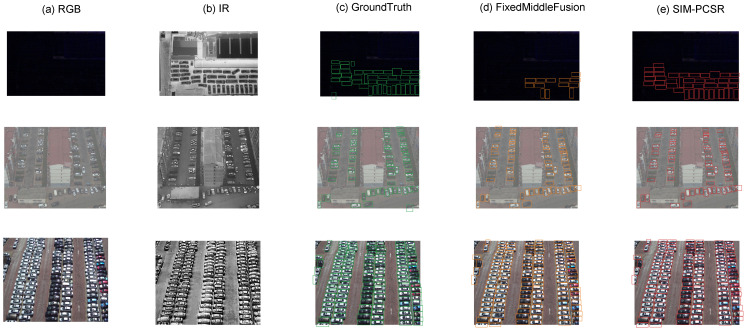
Qualitative comparison on representative DroneVehicle validation samples. Each row corresponds to one scene. The columns from left to right are (**a**) RGB image, (**b**) IR image, (**c**) ground truth, (**d**) Fixed Middle Fusion baseline, and (**e**) SIM-PCSR. The colored boxes denote annotated or detected objects. The three samples correspond to low illumination, normal visible-light response, and daytime dense-object scenes. Compared with the Fixed Middle Fusion baseline, SIM-PCSR maintains better object coverage under different imaging conditions, especially in dense small-object regions.

To provide a more intuitive analysis of the detection behavior, representative samples from the DroneVehicle validation set are selected for qualitative comparison, as shown in Figure 4. Each row corresponds to one scene, and the columns show the RGB image, IR image, ground truth, Fixed Middle Fusion baseline, and SIM-PCSR results. The selected samples cover low illumination, normal visible responses, and daytime dense parking scenes, reflecting typical difficulties in UAV RGB-IR detection, including dense small targets, modality contribution differences, and background interference.

The visualization shows that the Fixed Middle Fusion baseline still suffers from missed detections in several dense vehicle scenes, especially when objects are small, densely arranged, or locally affected by background interference. In comparison, SIM-PCSR detects more vehicles consistent with the ground truth, indicating that key-layer selective interaction and post-fusion complementary refinement help preserve cross-modal evidence that is useful for small objects. This observation is consistent with the positive gain on the small-object subset in the scale-wise analysis.

The samples also reflect different contributions of RGB and IR modalities. In low-light scenes, RGB textures are weak, while IR provides more stable vehicle contours. In normal illumination and daytime dense scenes, RGB already contains a richer appearance and structural information, but dense layouts and background interference still increase detection difficulty. Fixed Middle Fusion introduces dual-modal information, but without explicit selection and complementary reorganization, it may fail to sufficiently distinguish useful object responses from redundant background responses. SIM-PCSR uses prefiltering, selective response generation, and local window residual refinement to concentrate fused features on target regions with higher detection value, leading to more stable detection behavior under different modality contribution conditions.

The qualitative results of this study are not intended to replace quantitative evaluation. Instead, they provide visual evidence for the source of the improvement. Together with the main results, ablation studies, and further analyses, they show that the advantage of SIM-PCSR lies in selectively utilizing effective cross-modal information in complex scenes, rather than relying only on one modality or a specific lighting condition.

## 5. Discussion

The experimental results of this study show that the effectiveness of SIM-PCSR does not simply come from using dual-modal inputs. Instead, the improvement mainly comes from changing how cross-modal information is selected and utilized. In UAV RGB-IR small-object detection, RGB and IR modalities often contribute unequally. RGB images provide texture, color, and structural cues, but object details may degrade under low illumination, long-distance imaging, and complex backgrounds. IR images can provide more stable thermal responses, but they may also be affected by thermal background interference and dense object layouts. Therefore, direct concatenation or indiscriminate middle-layer fusion does not guarantee that complementary information will be effectively exploited. The gains achieved by SIM-PCSR indicate that selecting, organizing, and injecting modal evidence at the key layer is an effective way to alleviate modality imbalance and background interference.

The multi-seed evaluation further reveals an important metric-level difference. SIM-PCSR obtains a higher average mAP_50:95_ than the Fixed Middle Fusion baseline, while its average mAP_50_ is slightly lower. This phenomenon suggests that the proposed method does not mainly increase coarse detections under a loose IoU threshold. Instead, its benefit is more clearly reflected in the stricter overall detection quality measured across multiple IoU thresholds. Since mAP_50_ only evaluates whether predicted boxes roughly hit the targets at IoU = 0.50, it is less sensitive to localization quality. In contrast, mAP_50:95_ better reflects whether detection boxes are consistently accurate under progressively stricter localization requirements. This is consistent with the design goal of SIM-PCSR: the method aims to improve feature discriminability and object-region representation through key-layer complementary enhancement, rather than merely increasing the number of positive detections.

The scale-wise analysis and qualitative visualization also show that the effect of SIM-PCSR is not limited to a single object scale or a single imaging condition. Positive gains are observed for small, medium, and large objects, and the qualitative results show improved detection coverage in low-light scenes, normal visible-light scenes, and daytime dense-object scenes. These findings suggest that SIM-PCSR should be interpreted as a mechanism for improving the quality of key-layer cross-modal representation, rather than as a scale-specific post-processing strategy or a method that depends on one dominant modality. Although the method is motivated by small-object-sensitive P3 features, cleaner and more discriminative key-layer representations can propagate through the subsequent feature pyramid and benefit multi-scale detection.

Despite the effectiveness verified by the main comparison, module ablation, position ablation, complexity analysis, multi-seed evaluation, category-wise analysis, scale-wise analysis, and qualitative visualization, this work still has several limitations. First, the current experiments are mainly conducted on the DroneVehicle dataset. Although this benchmark provides paired UAV RGB-IR images and is suitable for evaluating the proposed setting, the generalization ability of SIM-PCSR under different sensor configurations, flight altitudes, weather conditions, and scene distributions still needs to be systematically evaluated on additional UAV RGB-IR datasets. Therefore, the results in this study should be interpreted as evidence on the DroneVehicle benchmark rather than as a complete validation across all UAV RGB-IR scenarios. Future work will further evaluate SIM-PCSR on additional UAV RGB-IR datasets and real UAV platforms to verify its cross-sensor and cross-scene generalization ability. Second, although SIM-PCSR achieves positive gains on small objects, the AP_50:95_ improvement for small objects remains modest because very small and dense targets are highly sensitive to localization errors under strict IoU thresholds. Further improving high-IoU localization for such targets remains an important direction. Third, the local window cross-attention and post-fusion residual refinement in PCSR introduce additional computational cost. Although the current inference speed remains acceptable on an RTX 4090 GPU, this does not directly represent performance on low-power embedded UAV hardware. Further optimization, including model compression, quantization, TensorRT acceleration, and lightweight attention design, is still needed for deployment scenarios with stricter real-time and power constraints. Finally, this work focuses on complementary enhancement at the P3 key layer. Future work may explore adaptive mechanisms that dynamically select enhancement layers according to object scale, scene complexity, or modality quality.

## 6. Conclusions

This paper addresses UAV RGB-IR small-object detection under modality imbalance, sparse object information, and background interference. To improve the utilization of cross-modal complementary information at the key layer, we proposed SIM-PCSR, a lightweight complementary enhancement method that decomposes cross-modal modeling into two stages: pre-fusion selective interaction and post-fusion complementary refinement. By concentrating the enhancement on the small-object-sensitive P3 layer, the proposed method improves key-layer representation while keeping the main detection framework unchanged.

Experiments on the DroneVehicle dataset demonstrate the effectiveness of the proposed method. Compared with representative single-modal and multimodal detection methods, SIM-PCSR achieves the best mAP_50_ and mAP_50:95_ in the main comparison. Module ablation and position ablation further confirm that pre-fusion selection, post-fusion reorganization, and the P3 key-layer setting all contribute to the final performance. Multi-seed evaluation, category-wise analysis, scale-wise analysis, and qualitative visualization also show that the improvement is not caused by single-run fluctuation or a single category, but is consistently reflected under stricter IoU evaluation, across multiple vehicle categories, and over different object scales.

Overall, SIM-PCSR provides a lightweight, pluggable, and problem-oriented key-layer complementary enhancement strategy for UAV RGB-IR small-object detection. Future work will further evaluate its generalization ability on more RGB-IR UAV datasets and under more complex scene conditions. More efficient local complementary modeling strategies will also be explored to reduce computational cost and improve deployment adaptability.

## Figures and Tables

**Table 1:** Main results on the DroneVehicle validation set.

Method	Modality	mAP_50_	mAP_50:95_	Params (M)	GFLOPs
Faster R-CNN [29]	RGB	72.428	47.221	43.277	450.815
RetinaNet [30]	RGB	50.218	33.001	36.415	164.183
YOLOv5s [31]	RGB	79.180	53.888	7.024	15.800
YOLOv8s [24]	RGB	78.581	54.922	11.138	28.700
Faster R-CNN [29]	IR	76.011	52.006	43.277	450.815
RetinaNet [30]	IR	60.199	41.127	36.415	164.183
YOLOv5s [31]	IR	82.112	58.897	7.024	15.800
YOLOv8s [24]	IR	82.592	60.068	11.138	28.700
DEYOLO [21]	RGB + IR	82.956	60.102	22.944	61.595
CFT [19]	RGB + IR	78.940	53.121	44.527	36.014
ICAFusion [20]	RGB + IR	82.032	59.603	23.256	30.130
SIM-PCSR (Ours)	RGB + IR	**85.323**	**63.572**	18.190	55.720

Bold values indicate the best results in the table.

**Table 2:** Module ablation of SIM-PCSR. A checkmark indicates that the corresponding component is used. TI denotes top-k selective interaction; RT denotes residual transfer to the fused P3 feature; RSP denotes remove–select prefiltering, including redundancy prediction and soft modality selection; WR denotes window-attention refinement.

Variant	TI	RT	RSP	WR	mAP_50_	mAP_50:95_
Baseline (Fixed fusion)					84.800	62.821
M1	✓				84.385	62.372
M2	✓	✓			85.008	62.962
M3	✓	✓	✓		85.034	62.508
M4	✓	✓		✓	85.293	63.197
SIM-PCSR (Full)	✓	✓	✓	✓	**85.323**	**63.572**

Bold values indicate the best results in the table.

**Table 3:** Position ablation of the SIM-PCSR refinement stage.

Variant	Insertion Position	mAP_50_	mAP_50:95_	Notes
Fixed Middle Fusion	No SIM-PCSR	84.800	62.821	Baseline without SIM-PCSR
SIM-PCSR-P5	P5	84.682	62.748	Refinement moved to P5
SIM-PCSR-P4	P4	84.264	62.825	Refinement moved to P4
SIM-PCSR	P3	**85.323**	**63.572**	Proposed key-layer setting

Bold values indicate the best results in the table.

**Table 4:** Accuracy–efficiency comparison between the baseline and SIM-PCSR.

Method	Params (M)	GFLOPs	Latency (ms/img)	FPS	mAP_50_	mAP_50:95_
Fixed Middle Fusion	17.03	43.66	12.54	79.76	84.800	62.821
SIM-PCSR	18.19	55.72	21.59	46.32	85.323	63.572

**Table 5:** Five-seed stability comparison. CI denotes the 95% confidence interval estimated using Student’s *t* distribution over five runs.

Method	mAP_50_ Mean ± std	mAP_50_ 95% CI	mAP_50:95_ Mean ± std	mAP_50:95_ 95% CI
Fixed Middle Fusion	84.943 ± 0.183	[84.716, 85.170]	62.738 ± 0.131	[62.575, 62.901]
SIM-PCSR	84.846 ± 0.308	[84.464, 85.228]	**63.071 ± 0.262**	**[62.745, 63.396]**

Bold values indicate the best result for each seed.

**Table 6:** Per-seed mAP_50:95_ results of Fixed Middle Fusion and SIM-PCSR.

Method	Seed 1	Seed 2	Seed 3	Seed 4	Seed 5
Fixed Middle Fusion	62.706	62.694	62.618	62.710	62.963
SIM-PCSR	**63.422**	**62.787**	**62.895**	**63.255**	**62.994**

Bold values indicate the best results in the table.

**Table 7:** Category-wise AP analysis on the DroneVehicle validation set.

Class	Instances	Fixed Middle Fusion mAP_50_	SIM-PCSR mAP_50_	Δ	Fixed Middle Fusion mAP_50:95_	SIM-PCSR mAP_50:95_	Δ
car	18,965	97.956	98.061	+0.105	73.391	73.462	+0.071
truck	1336	82.030	83.813	+1.783	58.958	60.450	+1.492
bus	751	97.397	97.291	−0.106	77.516	77.484	−0.032
van	700	73.221	73.935	+0.714	53.414	54.334	+0.920
freight car	710	72.869	73.472	+0.603	50.589	51.966	+1.377

**Table 8:** Scale-wise evaluation under the RGB-IR fused annotation protocol.

Method	AP_50_-Small	AP_50:95_-Small	AP_50_-Medium	AP_50:95_-Medium	AP_50_-Large	AP_50:95_-Large
Fixed Middle Fusion	26.141	13.344	82.469	60.241	61.373	48.893
SIM-PCSR	**27.136**	**13.756**	**83.075**	**60.890**	**62.562**	**50.809**
Δ	+0.995	+0.412	+0.607	+0.649	+1.189	+1.915

Bold values indicate the best results in the table.

## Data Availability

The DroneVehicle dataset used in this study is publicly available from its original providers. A minimal reproducible implementation, including the core modules, model configurations, dataset preparation script, and training templates, is provided as Supplementary Material for peer review. The source code will be publicly released at https://github.com/h1931397877/SIM-PCSR-Key-Layer-Complementary-Enhancement-for-UAV-RGB-IR-Small-Object-Detection (accessed on 16 May 2026) upon acceptance.

## Supplementary Materials

The following supporting information can be downloaded at: https://www.mdpi.com/article/10.3390/s26123806/s1, The supplementary code package submitted for peer review contains the minimal reproducible implementation of SIM-PCSR, including the core modules, model configurations, dataset preparation script, and training and validation templates.

## Abbreviations

The following abbreviations are used in this manuscript:

UAV	Unmanned aerial vehicle
RGB	Red–green–blue visible imaging modality
IR	Infrared imaging modality
RGB-IR	Visible–infrared multimodal imaging
HBB	Horizontal bounding box
OBB	Oriented bounding box
SIM	Selective interaction mechanism
PCSR	Prefiltering and Complementary Spatial Refinement
mAP	Mean average precision
GFLOPs	Giga floating-point operations
FPS	Frames per second
IoU	Intersection over union
C2f	Two-convolution cross-stage partial bottleneck
PAN-FPN	Path aggregation network–feature pyramid network
